# The controls that got out of control: How failed control experiments paved the way to transformative discoveries

**DOI:** 10.1038/s44319-025-00369-w

**Published:** 2025-01-20

**Authors:** André Schneider

**Affiliations:** Department of Chemistry, Biochemistry and Pharmaceutical Chemistry, University of Bern, Freiestrasse 3, CH-3012 Bern, Switzerland

**Keywords:** History & Philosophy of Science

## Abstract

When experimental controls go wrong, it does not necessarily mean your experiment is flawed. It might well be the first sign of something unexpected.

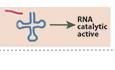

How would you define science to a lay person, considering that ‘science’ includes highly diverse disciplines? My answer would be that all these disciplines share a common foundation, called the ‘scientific method’. It provides a universally valid framework, enabling anyone, regardless of scientific or cultural background, to arrive at the same conclusions when analyzing the same data. Unlike faith, which is highly subjective, the scientific method offers an objective, systematic approach to uncover facts about the natural world.

In the life sciences, much progress has been driven by experimentation, a procedure by which researchers gather evidence that either supports or refutes a hypothesis. Crucial elements of every experiment are controls: reference points that connect new findings to previously established knowledge (Schickore, 2024). They put constraints on how you are allowed to interpret the experimental results. Without controls, experiments are essentially worthless, they lack scientific rigor and may result in misleading conclusions.

“Without controls, experiments are essentially worthless, they lack scientific rigor and may result in misleading conclusions.”

## The importance of controls

Controls reflect what has already and firmly been established in a field of research and therefore are generally boring. Moreover, controls can also be annoying. As everybody who ever submitted a manuscript to a scientific journal can testify, peer reviewers will almost invariably ask for additional control experiments, irrespectively of how many of them are already provided in the manuscript. However, when you are on the other side, acting as a reviewer, it becomes suddenly your job to assess the adequacy of experimental controls of other people’s studies. Everybody agrees that this scrutiny is essential for the peer-review process and, ultimately, the advancement of science.

Experimental controls fall into two main categories: negative controls and positive controls. Negative controls are expected to remain unaffected by the tested variables. They often involve the absence of a specific treatment or a crucial component, providing a baseline to assess whether any observed changes are truly caused by experimental intervention. Positive controls are more complicated. They apply a treatment that is known to produce an expected outcome consistent with the hypothesis. This helps to verify the validity of the experimental protocol and ensures that the experiment is functioning as intended.

If a control experiment fails, does that mean that the entire experiment is poorly designed and you must restart from scratch? Often, yes— but not always. I present five examples where failed control experiments triggered entirely new fields of research. In each case, the scientists initially approached the failed controls with great skepticism and thoroughly investigated possible trivial explanations. Yet, even though they found no plausible explanations why the controls failed, they chose not to abandon their projects. Instead, they embraced a mindset famously summarized by Sherlock Holmes: “Once you eliminate the impossible, whatever remains, no matter how improbable, must be the truth.” Sometimes, a failed control experiment can indicate that the well-established, widely accepted knowledge underlying it is wrong. In the examples discussed here, this was the case, and the failed controls led to groundbreaking discoveries, some of which resulted in paradigm shifts in science.

### RNA goes rogue

**Figure 1:**
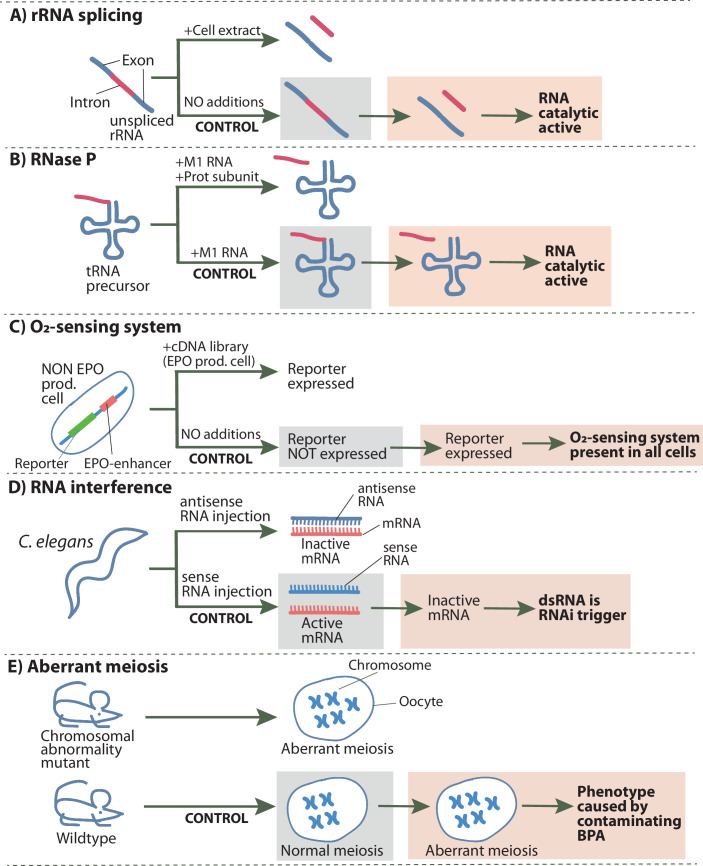
Groundbreaking discoveries initiated by failed control experiments. Schematic depiction of the key experiments described in the text where the experimental controls failed. The experimental treatments or the lack thereof are indicated on the top of each arrow. Top part of each panel depicts the actual experiment and its result. Bottom part of each panel depicts the failed control experiment (bold). The grey shaded box shows the expected result. On the left part of the red shaded box the actually obtained result is shown, whereas on the right part of the red shaded box the unexpected discovery, that was triggered by the failed control, is indicated in bold letters.

In 1978, Tom Cech started his own research group and used the extrachromosomal and massively amplified rDNA locus of *Tetrahymena*, which contains an intron, as a model system to study RNA splicing. To that end, his team isolated the unspliced precursor rRNA to characterize the splicing activity. However, things did not go as planned. In the in vitro assay, splicing occurred even in the negative control reaction in the absence of nuclear extract (Fig. 1A; Abelson, 2017). Initially, they suspected that the unspliced rRNA substrate might carry a tightly bound protein acting as a catalyst. But this was not the case, because even when they produced unspliced rRNA substrate by in vitro transcription, splicing still occured, proving that the splicing reaction must be independent of proteins (Kruger et al, 1982). They also discovered by which mechanism splicing occurred and showed that it required GTP as a cofactor. The monumental conclusion of these experiments: RNA can have catalytic activity!

Independently of the Cech group, Sydney Altmann came to similar conclusions. As a postdoc, Altmann had discovered RNase P, the enzyme that removes the 5’-extension of tRNA precursors. In 1978, after he had started his own lab, his group reported that purified RNase P from *E. coli* consisted of both a protein and a long RNA subunit, named M1 RNA. Treating RNase P with either RNase or proteinase inactivated it, demonstrating that both components were essential. Subsequently, they performed reconstitution experiments using in vitro transcribed M1 RNAs from *Escherichia coli* and *Bacillus subtilis* and the corresponding purified protein subunits. The negative control for these experiments aimed to confirm that M1 RNA alone was inactive. Surprisingly, however, the M1 RNAs were active on their own, provided that the Magnesium concentration was sufficiently high (Fig. 1B; Guerrier-Takada et al, 1983). Thus, the negative control turned into the experiment. Further analysis proved that M1 RNA functioned as a true enzyme: it remained unchanged during reactions, was required in small amounts only, and exhibited catalytic kinetics. The once-heretical idea that the catalytic activity of RNase P enzymes is mediated by RNA soon became the new dogma (Altman, 1990). An ironic consequence was that when the first protein-only RNase P in mammalian mitochondria was discovered in 2008, it was greeted with almost the same skepticism as the concept of catalytic RNA had been before.

The rest is history. The fact that RNA can both carry genetic information and act catalytically led to the RNA-world hypothesis: a plausible early stage in evolution where self-replicating RNA molecules did not depend on proteins or DNA. Furthermore, it was later shown that rRNA catalyzes ribosomal peptide-bond formation, arguably one of the most important biochemical reaction. It was no surprise therefore that Thomas R. Cech and Sydney Altman received the Nobel Prize in Chemistry in 1989 for their groundbreaking discovery of catalytic RNAs.

“The fact that RNA can both carry genetic information and act catalytically led to the RNA-world hypothesis: a plausible early stage in evolution where self-replicating RNA molecules did not depend on proteins or DNA.”

### Hold your breath

Oxidative phosphorylation is the primary source of ATP and hence energy in all animal cells—without oxygen, most non-photosynthetic organisms would die within a couple of minutes. Consequently, the increasing size of animals was enabled by the evolution of circulatory systems and oxygen-carrying erythrocytes since simple diffusion of oxygen could no longer reach all respiring cells. When oxygen levels drop, additional erythrocyte production is stimulated by erythropoietin (EPO), a protein hormone produced primarily in the kidneys and liver where oxygen-sensing cells trigger the production of EPO under low-oxygen conditions.

In the 1980s, scientists, including Peter Ratcliffe’s group, sought to uncover the molecular mechanisms of this oxygen-sensing system. They wanted to find out which cis-acting DNA sequences regulate oxygen-sensitive transcription of the EPO gene and identify the factors that bind to these sequences. Ratcliffe’s group used a hepatoma cell line known for its oxygen-sensitive EPO production and transfected these cells with variants of the EPO cDNA. This led to the discovery of a sequence downstream of the EPO gene that regulated oxygen-dependent transcription. This sequence acted as an enhancer and was recognized by specific transcription factors in hepatoma cells.

At the time, researchers—including Ratcliffe’s group—assumed that cells not producing EPO lacked this oxygen-sensing system. Based on this, in retrospect rather arbitrary, assumption Ratcliffe hypothesized that the factors involved in oxygen sensing could be identified through expression cloning. Practically, a cDNA library from hepatoma cells, which have the oxygen-sensing system, would be transfected into a non-EPO-producing cell line containing a reporter gene linked to the EPO enhancer. The obvious, but crucial, negative control for this approach was to show that expression of the reporter gene in the absence of the cDNA library is not regulated by oxygen.

However, they were in for a surprise: the reporter gene alone exhibited oxygen-sensitive activity in a variety of cell lines (Fig. 1C). At first, this unexpected result irritated Radcliffe, because it meant that there was no chance his elegant expression cloning approach could work. However, after some reflection he realized the exciting implications. The ‘failed’ negative control indicated that the oxygen-sensing system was not unique to EPO-producing cell, it was universal and likely regulated other cellular processes beyond erythropoiesis (Maxwell et al, 1993).

Using other experimental approaches eventually led to the identification of HIF-1 (hypoxia-inducible factor 1), the transcription factor that binds to the EPO enhancer and activates transcription under low-oxygen conditions. Today, we know how the oxygen-sensing system works. In a nutshell, it involves prolyl hydroxylase enzymes, which depend on molecular oxygen and hydroxylatespecific proline residues in HIF-1. When oxygen is plentiful, hydroxylation allows ubiquitination of HIF-1, marking it for degradation. However, at low-oxygen levels, hydroxylation is inhibited, stabilizing HIF-1, which then binds the enhancer and activates transcription of EPO.

What began with a failed negative control ultimately opened the door to understanding a universal oxygen-sensing mechanism that influences not only erythropoiesis but also numerous other processes such as metabolism, angiogenesis, cell survival, and immune responses. This groundbreaking work earned the 2019 Nobel Prize in Physiology or Medicine for Peter J. Ratcliffe, Gregg L. Semenza, and William G. Kaelin.

“What began with a failed negative control ultimately opened the door to understanding a universal oxygen-sensing mechanism that influences not only erythropoiesis but also numerous other processes…”

### Making sense of silencing

In 1995, Su Gou and Kenneth Kemphues conducted an antisense inhibition experiment in *C. elegans* by injecting in vitro transcribed antisense RNA targeting the *par-1* gene. Approximately 50% of the treated animals exhibited the embryonic lethal phenotype characteristic of a *par-1* loss-of-function mutation. Unexpectedly, injecting the corresponding sense RNA strand—the negative control experiment— produced the same result (Guo and Kemphues, 1995). The outcome was surprising, as antisense inhibition was expected to work by hybridization to the target mRNA (Fig. 1D). This failed control experiment stands out from the previous examples discussed above, because the authors published it despite its inconsistency with the prevailing model. While they maintained confidence in the antisense-assay based on other negative controls, they openly acknowledged their inability to explain why sense RNA was as effective as antisense RNA (Guo and Kemphues, 1995).

Researchers soon discovered that RNA interference (RNAi), a term later coined by Craig Mello, could be induced by both sense and antisense RNA and exhibited remarkable persistence. Previous studies had reported the production of small amounts of double-stranded RNA (dsRNA) during in vitro transcription. Based on this information, Andy Fire hypothesized that this contaminating dsRNA, which is more stable than single-stranded RNA, might be the trigger for RNAi (Fire, 2007).

In 1998, Fire and Mello’s groups tested this hypothesis. They demonstrated that purified single-stranded sense or antisense RNA alone had only background activity. However, when equal amounts of sense and antisense RNA were hybridized to form dsRNA, RNAi efficiency increased more than 100-fold. This groundbreaking discovery established dsRNA as the inducer of RNAi (Fire et al, 1998).

Today we know that RNAi is found in most eukaryotes. Its biological function is thought to be a defense mechanism against RNA viruses and transposable elements, both of which produce dsRNAs. Additionally, RNAi plays a significant role in gene regulation and has become a widely used tool for silencing specific mRNAs in various organisms. The details of the pathway have also been elucidated. In short, it is initiated by dsRNA precursors which are processed by the Dicer endonclease into short fragments of around 22 nucleotides, called small interfering RNAs (siRNAs). These siRNAs are incorporated into the RNA-induced silencing complex (RISC), where the two strands are separated. The guide strand remains bound to RISC and pairs with complementary mRNA sequences. This pairing activates the Argonaute protein within RISC, leading to mRNA cleavage and often to complete degradation of the target mRNA.

Thus, the groundbreaking discovery of RNAi was initiated by a failed control experiment demonstrating that the straightforward concept behind antisense inhbition could not explain the observed results. Intuition and out-of-the-box thinking subsequently revealed that the trigger of the process must be dsRNA. The efforts of Fire and Mello’s groups, alongside numerous others, uncovered the complexity of the RNAi mechanism. This discovery is another testament to how unexpected findings in science can lead to transformative breakthroughs. In 2006, Andrew Fire and Craig Mello were awarded the Nobel Prize in Physiology or Medicine for their discovery of RNAi as a gene-silencing mechanism mediated by dsRNA.

“… the groundbreaking discovery of RNAi was initiated by a failed control experiment demonstrating that the straightforward concept behind antisense inhbition could not explain the observed results.”

### The accidental sentinel

If meiosis, the process generating oocytes and sperm cells, goes wrong, it can produce cells with incorrect chromosome numbers, a leading cause of miscarriage and birth defects. Patricia Hunt’s group, studying the biology of infertility, analyzed transgenic mice mutants expected to show high chromosomal abnormalities. As negative controls, they used unmutated wild-type mice strains that should not exhibit such defects. Initially, the experiments went as expected: only 1.8% of control oocytes showed meiotic chromosome misalignments, resulting in 0.5–1% of hyperploid cells, containing supernumerary chromosomes. This was in the expected range.

However, in 1998, experiments comparing mutant and wild-type mice revealed an unexpected surge in chromosome misalignments and hyperploidy in the control mice (Hunt et al, 2003). During eight months, 9–40% of control oocytes displayed chromosome misalignments, averaging 5.8% of hyperploidy. These ‘negative’ controls were completely screwed up precluding meaningful comparisons with mutant mice. Subsequent investigations linked the meiotic disruptions to damaged polystyrene cages and drinking bottles, caused by the use of an incompatible cleaning detergent (Koehler et al, 2003). The damaged materials leached the polystyrene monomer bisphenol A (BPA).

Switching the mice to a different animals facility and the use polysulfone cages and glass bottles restored accurate meiosis. Further experiments confirmed that adding low concentrations of BPA to the drinking water, intended to mimic leaching from damaged cages, reproduced meiotic errors, though at lower levels than after accidental exposure. BPA is an estrogen mimic offering a plausible explanation for the oocyte abnormalities.

Thus, Hunt’s group directly linked short-time BPA exposure at concentrations considered safe for humans to disruption of female meiosis and thus reproductive health in mice. To what extent these results can be extended to humans is still unclear. However, just consider that the term microplastic, which was only coined in 2004, has since become as ubiquitous as the substance itself. Truly BPA-free organisms, critical as experimental controls, may no longer exist, which is an unsettling prospect for future studies (Landecker, 2013).

“… Hunt’s group directly linked short-time BPA exposure at concentrations considered safe for humans to disruption of female meiosis and thus reproductive health in mice.”

### Conclusions

How often do failed control experiments drive innovation? The five examples I chose highlight cases where the role of failed controls in the discovery process is explicitly documented in the scientific literature. Yet, standardized research articles typically aim to present results in a logical and coherent manner. As a result, discoveries are almost never reported in strict chronological order, and their precise history is often lost. However, anecdotal evidence based on numerous informal conversations I had at scientific meetings over many years suggests to me that failed control experiments that lead to intriguing new discoveries are more frequent than we might think.

Does this mean you should be excited if your negative control fails and gives you a positive result, and that you are on the verge of a groundbreaking discovery? A word of caution, most often than not, such unexpected results indicate a flaw in your experimental setup. But do not consider that as a failure: after all, uncovering potential problems is precisely the purpose of controls. That said, if the control consistently produces the ‘wrong’ result despite careful design and optimization of the experiment, you might be onto something significant. You may need to challenge some of the most basic assumptions you made when you designed your experiment. You should then turn your experiment on its head, convert the control into the experiment and explore the implications. It is possible that you have proceeded into an unknown corner of your research discipline. – Good luck!

“That said, if the control consistently produces the ‘wrong’ result despite careful design and optimization of the experiment, you might be onto something significant.”

## Supplementary information

Peer Review File

## References

[CR1] Abelson J (2017) The discovery of catalytic RNA. Nat Rev Mol Cell Biol 18(11):653. 10.1038/nrm.2017.105 29018284 10.1038/nrm.2017.105

[CR2] Altman S (1990) Nobel lecture. Enzymatic cleavage of RNA by RNA. Biosci Rep 10(4):317–37. 10.1007/BF01117232 1701103 10.1007/BF01117232

[CR3] Fire A, Xu S, Montgomery MK, Kostas SA, Driver SE, Mello CC (1998) Potent and specific genetic interference by double-stranded RNA in *Caenorhabditis elegans*. Nature 391(6669):806–11. 10.1038/35888 9486653 10.1038/35888

[CR4] Fire AZ (2007) Gene silencing by double-stranded RNA (Nobel Lecture). Angew Chem Int Ed 46(37):6966–84. 10.1002/anie.200701979 10.1002/anie.200701979 17722137

[CR5] Guerrier-Takada C, Gardiner K, Marsh T, Pace N, Altman S (1983) The RNA moiety of ribonuclease P is the catalytic subunit of the enzyme. Cell 35(3 Pt 2):849–57. 10.1016/0092-8674(83)90117-4 6197186 10.1016/0092-8674(83)90117-4

[CR6] Guo S, Kemphues KJ (1995) par-1, a gene required for establishing polarity in *C. elegans* embryos, encodes a putative Ser/Thr kinase that is asymmetrically distributed. Cell 81(4):611–20. 10.1016/0092-8674(95)90082-9 7758115 10.1016/0092-8674(95)90082-9

[CR7] Hunt PA, Koehler KE, Susiarjo M, Hodges CA, Ilagan A, Voigt RC et al (2003) Bisphenol a exposure causes meiotic aneuploidy in the female mouse. Curr Biol 13(7):546–53. 10.1016/s0960-9822(03)00189-1 12676084 10.1016/s0960-9822(03)00189-1

[CR8] Koehler KE, Voigt RC, Thomas S, Lamb B, Urban C, Hassold T et al (2003) When disaster strikes: rethinking caging materials. Lab Anim 32(4):24–7. 10.1038/laban0403-24 10.1038/laban0403-24 19753748

[CR9] Kruger K, Grabowski PJ, Zaug AJ, Sands J, Gottschling DE, Cech TR (1982) Self-splicing RNA: autoexcision and autocyclization of the ribosomal RNA intervening sequence of Tetrahymena. Cell 31(1):147–57. 10.1016/0092-8674(82)90414-7 6297745 10.1016/0092-8674(82)90414-7

[CR10] Landecker H (2013) When the control becomes the experiment. Limn 3:6–8

[CR11] Maxwell PH, Pugh CW, Ratcliffe PJ (1993) Inducible operation of the erythropoietin 3’ enhancer in multiple cell lines: evidence for a widespread oxygen-sensing mechanism. Proc Natl Acad Sci USA 90(6):2423–7. 10.1073/pnas.90.6.2423 8460154 10.1073/pnas.90.6.2423 PMC46099

[CR12] Schickore J (2024) Introduction: Practices, strategies, and methodologies of experimental control in historical perspective. Archimedes 71:2–19. 10.1007/978-3-031-52954-2_1

