# Supplementary material for: The controls that got out of control: How failed control experiments paved the way to transformative discoveries
Source: EMBO Rep. 2025 Jan 20;26(3):597–601. doi: 10.1038/s44319-025-00369-w (PMC11811022; doi:10.1038/s44319-025-00369-w)
Supplement: Supplementary file 1 — Peer Review File [file 44319_2025_369_MOESM1_ESM.pdf]

EMBO reports

The controls that got out of control

André Schneider

Corresponding author(s): André Schneider (andre.schneider@unibe.ch)

Review Timeline:

Submission Date:	13th Dec 24
Accepted:	9th Jan 25

Editor: Holger Breithaupt

Transaction Report: This article was editorially reviewed.
